# Unveiling the spectrum: A cross-sectional exploration of hirsutism causes in women

**DOI:** 10.12669/pjms.40.4.8271

**Published:** 2024

**Authors:** Rana Tabassum Ansari, Uneeba Syed, Musarrat Riaz, Saima Askari, Sarwat Anjum

**Affiliations:** 1 Rana Tabassum Ansari, FCPS. Fellow Endocrine, Baqai Institute of Diabetology and Endocrinology, Baqai Medical University, Karachi, Pakistan; 2 Uneeba Syed, FCPS. Assistant Professor, Consultant Endocrinologist, Allama Iqbal Medical College, Jinnah hospital, Lahore, Pakistan; 3 Musarrat Riaz, FCPS. Department of Medicine, Associate Professor, Consultant Endocrinologist, Baqai Institute of Diabetology and Endocrinology, Baqai Medical University, Karachi, Pakistan; 4 Saima Askari, FCPS. Department of Endocrinology/Medicine, Assistant Professor, Consultant Endocrinologist, Baqai Institute of Diabetology and Endocrinology, Baqai Medical University, Karachi, Pakistan; 5 Sarwat Anjum, FCPS. Consultant Endocrinologist, Baqai Institute of Diabetology and Endocrinology, Baqai Medical University, Karachi, Pakistan

**Keywords:** Congenital Adrenal Hyperplasia, Hirsutism, Hyperandrogenism, Polycystic ovaries, Ferriman-Gallwey score

## Abstract

**Background and Objective:**

Hirsutism is a common endocrine disorder and its etiology varies from benign and idiopathic disorders to serious malignant diseases. Hirsutism creates negative impact on quality of life and considerable effects on fertility. Our objective was to determine the various causes of hirsutism in women presenting at two endocrine clinics.

**Method:**

This cross-sectional study was conducted at Baqai Institute of Diabetology and Endocrinology, Karachi and at Jinnah hospital, Lahore from August 2020 to December 2021 women between 12-45 years of age with complains of hirsutism were included in the study. Severity of Hirsutism was evaluated using modified Ferriman-Gallwey score (FG). Patients with modified FG score of 8 or more were considered having hirsutism.

**Results:**

The study had 113 patients with a mean age of 15.50+7.29 years with 89% having moderate hirsutism (FG score 16-25). Polycystic ovaries was the most common cause of hirsutism. Common sites for hirsutism included back (83%), arms (74%), buttocks (70%), and upper abdomen (47%). High BMI (p-value <0.01) and high Dehydroepiandrosterone levels were positively associated with the severity of hirsutism (p-value of 0.006.)

**Conclusion:**

The various causes of hirsutism identified were polycystic ovaries, followed by idiopathic, thyroid dysfunction, congenital adrenal hyperplasia, and hyperprolactinemia; therefore, all women presenting with hirsutism should be evaluated for potential serious and curable etiologies, before embarking on a treatment plan.

## INTRODUCTION

Hirsutism is the term used to describe excess terminal hair growth in females in androgen-dependent areas of the body in a pattern identical to male, affecting up to 8% of women.^1^ Characteristically, it is associated with hyperandrogenemia. Most common causes of hirsutism are polycystic ovarian syndrome (PCOS) 57.7%, followed by idiopathic hirsutism 22.6%. Other causes include late onset congenital adrenal hyperplasia (CAH) 9.9%, hyperprolactinemia and thyroid disorders 4.2%. Whereas, pituitary, ovarian, and adrenal tumors are rare causes of hirsutism.^2^,^3^ Hirsutism may also be associated with obesity, insulin resistance, diabetes, hypertension, infertility, and menstrual irregularities. In Pakistan, the frequency of hirsutism is reported in 30% sub fertile females.^4^

Hirsutism, in most cases, is a relatively benign condition.^2^ Regardless of etiology it causes considerable emotional and psychological distress.^5^ Identification of serious underlying disorders is the primary purpose of laboratory testing and should be individualized. About 95% of these patients have PCOS or idiopathic hirsutism.^6^,^7^ Idiopathic hirsutism, is a term used in eumenorrheic women, who have no other clinical evidence suggesting PCOS or other hyperandrogenic endocrine disorder and is responsible for 5% to 20% cases, although some may have polycystic ovary morphology on ultrasound.^8^ Adrenal and ovarian neoplasms are responsible for severe cases of hirsutism.

Hirsutism severity varies according to androgen levels, it may be affected by the sensitivity of hair follicles to androgens.^6^ In females, the key role of androgen is to act on the body’s sex-specific areas and transform small, straight, fair vellus hairs into larger, curlier, and darker terminal hairs.^9^ The key factors involved in the growth and development of sexual hair include Androgens such as testosterone (T), dihydrotestosterone, and prohormones like dehydroepiandrosterone sulfate (DHEAS), and androstenedione.^10^ Hyperandrogenemia apart from hirsutism can also exhibit as acne, menstrual dysfunction, alopecia, or it could be asymptomatic.^6^

Hirsutism classification is based on the degree of growth and distribution of hair, the most used tool for this purpose is the Ferriman-Gallwey scale.^10^ Androgen-dependent growth areas affected include the upper lip, chin, chest, upper and lower abdomen, upper arms, thighs, upper and lower back. This may be associated with other signs of virilization, including temporal balding, masculine habitus, deepening voice, clitoral hypertrophy, and amenorrhea.^11^

Hirsutism negatively influences psychological well-being, especially in young women. Diagnosis of Hirsutism is made on clinical basis as the presence of hirsutism is a potential indication of an underlying hyperandrogenic disorder that may require specific treatment and may have distinct implications for fertility, and medical risks.^5^

Hirsutism is often perceived as a cosmetic concern rather than a medically significant condition with potential serious health consequences. The purpose is to shed light on the health implications linked to hirsutism, emphasizing the need to raise awareness among individuals at risk. Hence our study aimed to determine the various causes of hirsutism in patients attending endocrine clinics at two major hospitals in Karachi and Lahore.

## METHODS

This cross-sectional study was conducted at Baqai Institute of Diabetology and Endocrinology (BIDE) Karachi and Jinnah Hospital, Lahore from August 2020 to December 2021. A written informed consent was taken from participants prior to data collection and all the information acquired was kept confidential.

### Ethical approval

It was obtained from BIDE prior to data collection with approval# BIDE/IRB/RTANSARI/08/20/20/0239.

***Inclusion criteria*** consists of all women between the ages of 12 to 45 years, with hirsutism (as assessed by Ferriman & Gallway score) presenting at the endocrinology clinic, were recruited by using non-probability consecutive sampling. Pregnant, & postmenopausal females were excluded from the study. A sample size of 113 was calculated using open Epi. Taking 8%^12^ prevalence of hirsutism with 95% confidence level with 0.05 margin of error.

Hirsutism severity was assessed by using modified Ferriman & Gallway score.^13^ A cutoff of 8 and more was set for the diagnosis of hirsutism, score 8 to 15 considered as mild, 16 to 25 as moderate and more than 25 score labelled as severe hirsutism.

Laboratory investigations were based upon clinical history and examination and included serum Follicle Stimulating Hormone (FSH), Luteinizing Hormone (LH), 17-hydroxy progesterone (17-OHP), Testosterone, Thyroid Stimulating Hormone (TSH), fasting serum insulin, fasting blood glucose/HbA1c in all patients and Dexamethasone suppression test, Dehydroepiandrosterone sulphate (DHEAS) where indicated.

Imaging included ultrasound pelvis for Polycystic ovaries as well as Computed tomography of abdomen where required.

### Operational definitions:

PCOs, characterized by hyperandrogenism menstrual irregularities and polycystic ovarian morphology, diagnosed based on Revised Rotterdam Criteria.

PCAH, a group of genetic disorders affecting the adrenal glands, resulting in abnormal production of cortisol and/or excess androgens.

Hyperprolactinemia, elevated levels of prolactin in blood more than normal.

### Thyroid Disorders

hypothyroidism or hyperthyroidism were determined by deviation from normal range of TSH and T_4._

Idiopathic, where no cause was determined.

### Statistical analysis

All the acquired data was analyzed by using SPSS version 20. Descriptive statistics were reported in the form of mean and standard deviation for quantitative, and frequencies and percentages for qualitative variables. Chi-square test was used to determine the association between biochemical parameter and severity of hirsutism, at p-value<0.05 considered as significant.

## RESULTS

A total of 113 women with a mean age of 15.50+7.29 years were included in the study. The mean age of menarche was found 12.77+1.07years. Irregular menstrual cycle was reported by 77% (n7), with 13.2% (n22) had experienced amenorrhea for more than six months, 56.6% (n64) had oligomenorrhea, and 13.2% (n15) had a history of both amenorrhea and oligomenorrhea. Furthermore, 4.9% (n5) had a family history of hirsutism. The details of demographic characteristics and clinical presentation of hirsutism patients are reported in Table-I.

**Table-I:** Demographic Characteristics of Participants.

Variable	categories	N	%
Age	12-21	40	35.4
	22-31	48	42.5
	32-41	23	20.4
	42-45	2	1.8
Age of Menarche	11	11	9.7
	12	36	31.9
	13	46	40.7
	14	8	7.1
	15	12	10.6
BMI (Kg/m^2^)	<18.5	4	3.5
	18.5-24.9	27	23.9
	25-29.9	27	23.9
	30->30	55	48.7
Menstrual Irregularity	Yes	87	77.0
	No	26	23.0
Hypertension	Yes	26	23.0
	No	87	77.0
Diabetes	Yes	14	12.4
	No	99	87.6
Dyslipidemia	Yes	63	55.8
	No	50	44.2

The overall scoring of Ferrimen-Gallwey severity of hirsutism showed majority 89.3% (n101) of the patients were having moderate level of severity with scores ranging 16-25. Higher frequencies of hair growth were reported at back 83% (n94), arms 74.3% (n84), and buttocks 69.9% (n79), and upper abdomen regions 46.9% (n53).

**Fig.1:**
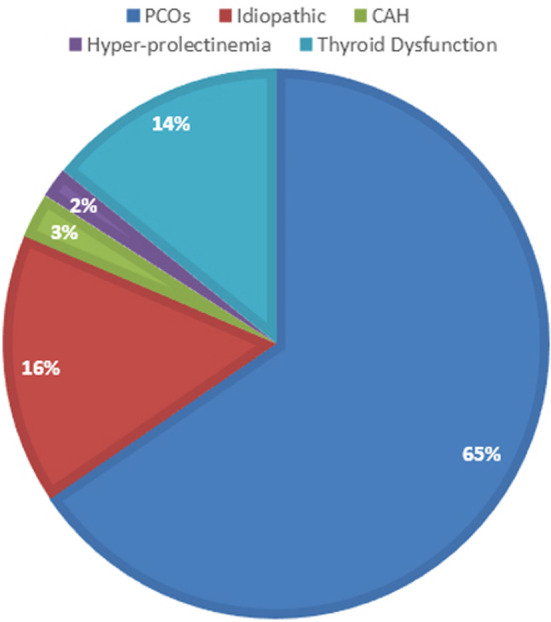
Causes of hirsutism.

The frequencies and percentage of etiologies of hirsutism patients were 65% (n74) had PCOs, 16% (n18) had idiopathic hirsutism, 14% (n16) were with thyroid dysfunction, 2.6% (n3) were diagnosed with CAH and 1.7% (n2) were diagnosed with prolactinoma (Fig.1).

Association of etiology with hirsutism severity showed no significant association (Table-II). Similarly, association of BMI with etiology of hirsutism showed that hirsutism most frequently affected obese patients of PCOs, CAH and Thyroid dysfunction. Etiology of hirsutism was found significant associated BMI categories with p-value <0.01.

**Table-II:** Severity of Hirsutism Crosstabulation with Etiology.

		Etiology					Total

Total Score on Ferriman-Gallwey Hirsutism Scoring System		PCOs	Idiopathic	CAH	Hyper-prolectinemia	Thyroid Dysfunction	
Mild (8-15)	N	2	0	0	0	0	2
	%	100	-	-	-	-	100
Moderate (16-25)	N	66	15	3	2	15	101
	%	65.3	14.9	3	2	14.9	100
Severe (>25)	N	6	3	0	0	1	10
	%	60	30			10	100
Total	N	74	18	3	2	16	113
	%	65.5	15.9	2.7	1.8	14.6	100

The hormonal profile varied with the underlying pathology. Association between biochemical parameters showed that high frequencies were reported within the normal ranges of biochemical parameters levels. However, irrespective of biochemical parameters levels majority of hirsutism severity frequencies were observed in moderate category. DHEA have also showed a strong association with the severity of hirsutism with p-value 0.006 (Table-III).

**Table-III:** The Biochemical Parameter in Women with Hirsutism

Biochemical Markers	Level of Marker	Severity Of Hirsutism			χ^2^	p-value

		Mild	Moderate	Severe		
Fasting Insulin	Normal	1	43	4	0.72	0.965
	Increased	1	58	6		
Estradiol	Normal	2	101	10	-	-
	Increased	-	-	-		
DHEA	Normal	1	97	10	10.34	0.006
	Increased	1	4	0		
Testosterone	Normal	0	11	2	0.964	0.617
	Increased	2	88	8		
FSH	Normal	2	92	10	1.162	0.559
	Increased	0	9	0		
LH	Normal	2	98	10	0.366	0.833
	Increased	0	3	0		
Progesterone	Normal	1	38	4	0.082	0.960
	Increased	1	57	6		
Serum Prolactin	Normal	1	51	4	0.906	0.989
	Increased	1	47	6		
Thyroid	Normal	2	86	9	0.512	0.774
	Increased	0	15	1		

## DISCUSSION

In the current study, we have attempted to ascertain the predominant causes of hirsutism presenting at the study sites. The demographic analysis reveals that hirsutism is more prevalent among adolescent (15.50+7.29 years) in our population, which is similar to the finding reported by Bangladesh.^12^ However, other southeast Asia studies has reported a relatively older population affected by hirsutism i.e., 24 years and above.^13^,^14^

In our study the most common cause of hirsutism was polycystic ovaries (PCOs), followed by idiopathic, thyroid dysfunction, CAH, and hyperprolactinemia. Similar trends were reported by earlier studies, where PCOS has been identified as the leading cause followed by idiopathic hirsutism.^12^,^15^-^18^ However, etiologies like non-classic congenital adrenal hyperplasia, androgen-secreting tumors, medications, hyperprolactinemia, thyroid disorders, and Cushing syndrome varied among different population.^19^,^20^

Common clinical findings of the study revealed that majority patients experienced irregular menstrual cycles, and dyslipidemia, which was present in more than half of the women. Weight gain was reported in the majority of patients, with half of the patients falling in either class-I, or class-II obesity and a strong association of hirsutism with the BMI was reported. Hirsutism was more common in patients with increased BMI. Similar findings of 51% were reported in a study from Saudi Arabia.^21^

In this study mostly women were moderately hirsute, having score between 16-25 on Ferriman-Gallwey scoring. Most common body area affected by hirsutism were back, arms, buttocks, and upper abdomen. However, prior studies reported mild to moderate hirsutism and the most affected areas were face, chin, and upper lips.^22^ Hirsutism is the most distressing dermatological manifestation of PCOS. Studies have shown that ethnic variations in the rate of hair growth should be considered in all patients of hirsutism.^23^,^24^ Association of etiology with severity of hirsutism showed no significant difference as most participants were moderately hirsute irrespective of the cause. A Previous study done in Karachi reported varying degree of hirsutism in 47.36% of patients.^4^

In our study, the examination of biochemical markers revealed that most of the biochemical markers were within normal range; except, testosterone progesterone, 17OHP, and prolactin which were elevated. Association of biochemical parameter with severity of hirsutism showed that DHEA has a significant association with severity of hirsutism as patients with higher level of DHEA manifest severe hirsutism.^24^

### Limitations

The main limitation of this study pertains to the relatively small sample size, because study was confined to only two endocrine centers in Pakistan. Furthermore, the exclusive enrollment of hospital-based presenting patients introduces a potential source of bias, limiting the generalizability of our findings to the broader population. To enhance the robustness and external validity of future investigations, we advocate for an expansion in both the scale and scope of the study.

## CONCLUSION

The various causes of hirsutism were polycystic ovaries, idiopathic, thyroid dysfunction, congenital adrenal hyperplasia, and hyperprolactinemia; with PCOs being the most common cause of hirsutism among the given population.

### Recommendation

We recommend that patients with hirsutism should be evaluated thoroughly for underlying potentially curable etiologies before initiating the treatment.

### Authors Contribution:

**RTA:** Design, Literature search, interpretation of data, wrote the manuscript.

**US:** Literature search, Interpretation of data, prepared the manuscript.

**MR:** Concept, design, involved in quality control, edited, and approved the final manuscript.

**SA & SA:** Involved in quality control, edited, and approved the final manuscript.

All authors are accountable for the integrity of work.
